# Spatial distribution and determinants of unmet need for family planning among all reproductive‑age women in Uganda: a multi‑level logistic regression modeling approach and spatial analysis

**DOI:** 10.1186/s40834-024-00264-0

**Published:** 2024-02-02

**Authors:** Alemayehu Sayih Belay, Haribondhu Sarma, Gizachew Yilak

**Affiliations:** 1 College of Medicine and Health Sciences, Department of Nursing, Wolkite University, P.O. Box: 07, Wolkite, Ethiopia; 2 National Centre for Epidemiology and Population Health, Colleague of Health and Medicine, The Australian National University, Canberra, ACT 2601 Australia; 3 College of Medicine and Health Sciences, Department of Nursing, Woldia University, P.O. Box: 400, Woldia, Ethiopia

**Keywords:** Unmet need, Women, Uganda, Spatial Analysis, Multi-level logistic analysis

## Abstract

**Introduction:**

Unmet need for family planning is defined as the percentage of sexually active and fecund women who want to delay the next birth (birth spacing) or who want to stop childbirth (birth limiting) beyond two years but who are not using any modern or traditional method of contraception. Despite the provision of family planning services, the unmet need of family planning remains a challenge in low- and middle-income countries (LMICs). Thus, this study aimed to assess the spatial distribution and determinant factors of unmet need for family planning among all reproductive‑age women in Uganda.

**Methods:**

A secondary data analysis was done based on 2016 Ugandan Demographic and Health Surveys (UDHS). Total weighted samples of 18,506 women were included. Data processing and analysis were performed using SPSS Version 26, STATA 14.2, ArcGIS 10.8, and SaTScan 10.1.2 software. Spatial autocorrelation and hotspot analysis was made using Global Moran’s index (Moran’s I) and Gettis-OrdGi*statistics, respectively. Determinants of unmet needs for family planning were identified by multi-level logistic regression analysis. Variables with a *p*-value < 0.05 were declared statistically significant predictors.

**Results:**

The spatial distribution of unmet need for family planning among women of reproductive age in Uganda was found to be clustered (Global Moran’s I = 0.27, Z-score of 12.71, and *p*-value < 0.0001). In the multivariable multilevel logistic regression analysis; women in West Nile (AOR = 1.86, 95% CI: 1.39, 2.47), aged 25–49 years old (AOR = .84; 95% CI .72, .99), highly educated (AOR = .69; 95% CI .54, .88), Muslim (AOR = 1.20, 95% CI: 1.03, 1.39), high wealth status (AOR = .73, 95% CI: .64, .82), and had five or more living child (AOR = 1.69, 95% CI: 1.51, 1.88) were significant predictors of unmet need for family planning. Significant hotspot areas were identified in West Nile, Acholi, Teso, and Busoga regions.

**Conclusion:**

A significant clustering of unmet need for family planning were found in Uganda. Moreover, age, educational status, religion, wealth status, number of alive children, and region were significant predictors of unmet need for family planning. Therefore, in order to minimize the burdens associated with unmet need, an interventions focusing on promotion of sexual and reproductive health service should be addressed to the identified hotspot areas.

## Introduction

Family planning (FP) is considered as one of the major public health concern, worldwide. Family planning has numerous social, economical, and health benefits for women. It prevents unintended pregnancies which in turn can also reduce the economic burden, risk of unsafe abortions, and other feto-maternal complications [1]. Unmet need for family planning is defined as the percentage of sexually active and fecund women who want to delay the next birth (birth spacing) or who want to stop childbirth (birth limiting) beyond two years but who are not using any modern or traditional method of contraception [2, 3].

Worldwide in 2019, among 1.9 billion women of reproductive age (WRA) group (15–49 years) [4]; an estimated 874 million, 92 million women use a modern contraceptive method and a traditional contraceptive method, respectively, whereas, 164 million have an unmet need for contraception and the remaining 807 million women of reproductive age group have no need for family planning [5]. The unmet need for family planning and the ineffective use of a contraceptive method can contribute to 121 million unintended pregnancies annually – accounts 48 percent of all pregnancies [6].

Globally, of all unintended pregnancies, 61 percent end in abortion, that is, 73 million abortions annually [6]. An estimated 45 percent of all abortions are unsafe [7] which causes for 38,940 deaths every year and most of these deaths are concentrated in low-income countries (LICs) [8]. Unintended pregnancy can also contribute to different temporary and permanent adverse impacts on maternal, child and family lives, including socioeconomic deprivation, suicidal ideation, depression [9], low birth weight (LBW) and preterm labor [10], and complications from unsafe abortions, among others [11].

Despite the global estimate of the proportion of the unmet need for modern methods declined from 17.6% in 1990 to 14.2% in 2019, the prevalence of unmet need for family planning in east Africa remains high, 24.66% [12]. Even though different strategies by International Conference on Population and Development (ICPD + 5) [13] and United Nations Population Fund (UNFPA) Strategy for Family Planning [14] were developed for the reduction of the burden of unmet need for family planning, still in Africa one from five women had unmet need for family planning [15]. Unmet need for family planning remains very high in different countries of sub-Saharan Africa like Liberia 35.9% [16], Uganda 28% [17], Ethiopia 22% [18], Gambia 17.9% [19], Burundi 32.4% [20], Egypt 16.28% [21], and Botswana 9.6% [22].

Unmet need for family planning has different contributing factors such as; low paternal educational status [23–25], having more children [26–28], young maternal age [20, 22, 23, 29], women have no any media exposure [26–28], infrequent home visit by FP workers [24, 29], married women [20, 29], engaged in unskilled work [26–28], women religion of being non-Christians [22, 29], region with low infrastructure [19, 20], low occupational status [23, 25, 29], and low household economic status [20, 25, 29].

Furthermore, various studies also revealed that poor decision-making ability of women [30–32], history of not using of FP [33], multiparity [19, 23, 25], partner’s non-supportive attitude towards family planning [22, 24], poor knowledge on the current menstrual status [24], being married more than once [30–32], poor discussion with partner about FP [16, 22–24], and husbands desire other child [19, 20] were the factors which increases the odds for the unmet need for family planning.

Particularly in Uganda, persistently high fertility rate has been attributed to young age at first marriage and low levels of contraception among women [34]. In contrast to this, women with some socio-demographic characteristics like advanced educational status and urban residency were contributed for the declining of fertility rates [35]. Another study conducted in Uganda also revealed that factors like; education, prior use of contraceptives, and partner communication about contraceptives were predictors of uptake of family planning services and contraceptive use [36]. Moreover, the proportion of modern FP users was considerably higher among mothers of children aged 7–12 months and urban residents compared to those mothers of children aged 0–6 months and rural residents, respectively [37].

According to the National Population and Housing Census (NPHC) report of Uganda, the unwanted teenage pregnancy and maternal death has been attributed to lack of, or limited access by adolescent girls to FP, education, and services. This could also lead to high levels of unsafe abortions, and maternal morbidity and mortality [38].

Following the London Summit on family planning in 2012, the government of Uganda has committed to universal access to family planning method to reduce unmet need for family planning from 40 to 10 percent in 2022 [39]. However, the annual budget for family planning supplies was also increased to achieve this goal; the estimated magnitude of unmet need for family planning among married women was 29.7% in 2022 [40]. Despite the some achievements towards reducing the unmet need for family planning were made, the country is still far from achieving the National FP targets. This is due to poor multi-sectoral collaboration and presence of suboptimal leadership responsibility and authority towards repositioning of family planning at subnational level [39].

Besides, the changing fertility intentions of women and inadequate availability of service delivery points could also be a challenge for achieving met need for FP [41]. Therefore, assessing for the spatial distribution and determinants of unmet need for family planning will be helpful to identify the different contributing factors of unmet need for FP and to achieve national goal and Sustainable Developmental Goals (SDGs) [42].

Moreover, study on the spatial distribution and determinants of unmet need for family planning help the policymakers and different health officers to design appropriate policy, to assess the health system disparities across different regions, to distribute information regarding family planning methods through different mass-medias, and to initiate different multi-sectoral collaboration for the reduction of the burden of unmet need for family planning (unintended pregnancy, school drop-out, and among others).

In Uganda, different studies regarding unmet need for family planning were conducted previously focusing on the individual-level analysis however multilevel analysis and spatial analyses regarding the unmet need for family planning have not been conducted to identify areas with hotspots. But in this study, to check for the cluster effect and identify areas with hotspots, multilevel analysis and spatial analysis on unmet need for family planning was done among women of reproductive age in Uganda, respectively. Therefore, this study aimed to assess the spatial distribution and determinant factors of unmet need for family planning among reproductive age women in Uganda.

## Methods

### Study design, data source and setting

An in-depth analysis of the Uganda Demographic and Health Survey (UDHS) 2016 data was used. The UDHS 2016 data was downloaded from the DHS program website (https://dhsprogram.com/data/dataset_admin/index.cfm/) after permission was granted. The cross-sectional study design was used to conduct the sixth national population-based survey (2016 UDHS). This study was conducted in Uganda, located in southeast Africa between 1º S and 4º N latitude, and between 30º E and 35º E longitude [43].

**Fig. 1:**
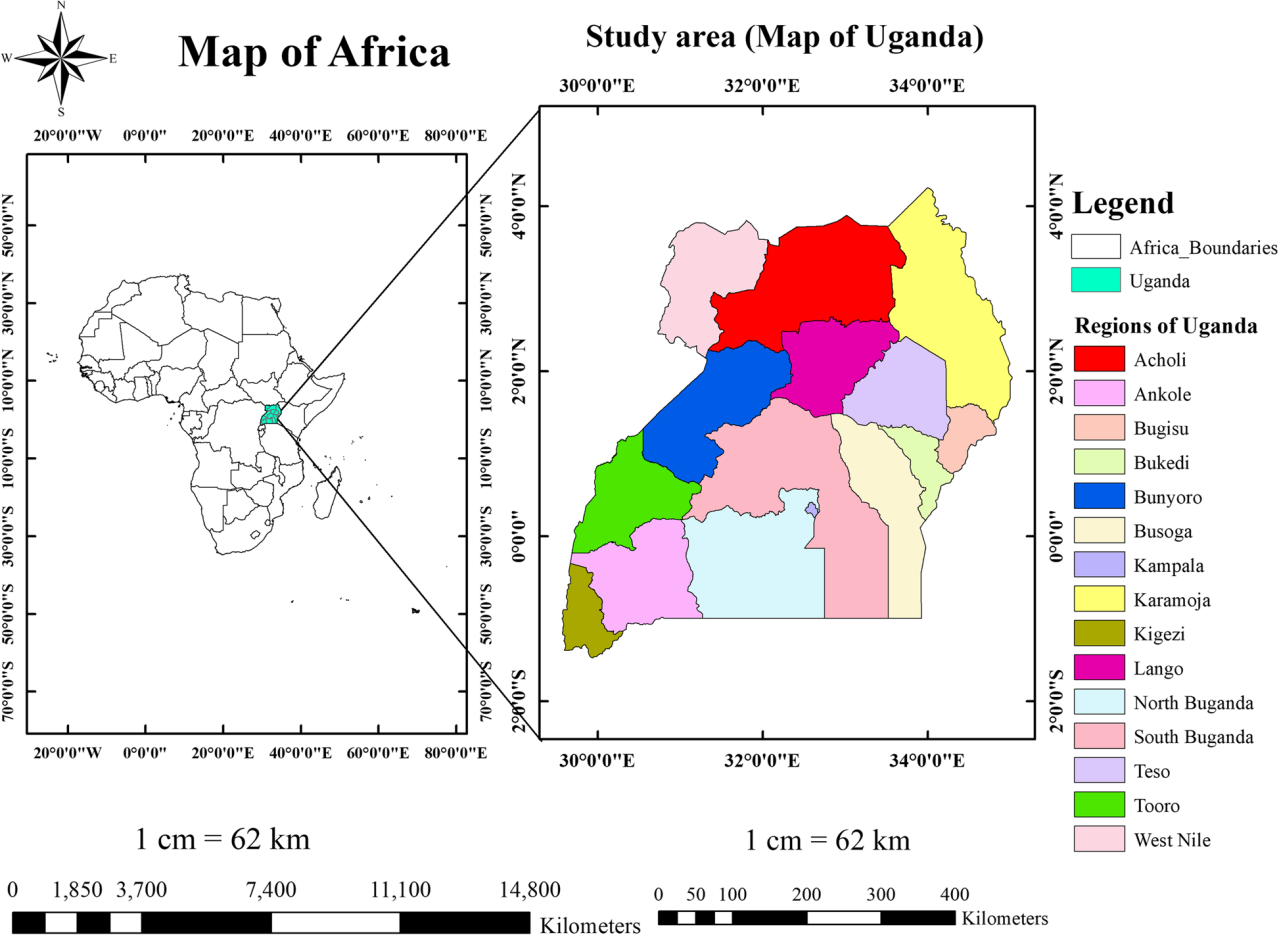
Map of the study area (Uganda) for spatial distribution and determinants of unmet need for family planning among women of reproductive age, Uganda, 2016

The country sits at an average of 900 m above sea level. According to the world-bank report in 2020, the country has a total area of 200,520.0 square Km [44]. Uganda is divided into four administrative regions. These regions further  divided into fifteen sub-regions (Central regions (Kampala, North Buganda, and South Buganda), Eastern regions (Bukedi, Bugisu, Busoga, and Teso), Western regions (Tooro, Ankole, Bunyoro, and Kigezi), and Northern regions (Acholi, Karamoja, Lango, and West Nile)) [17, 45] **(**Fig. 1**)**.

#### Study population and sampling procedure

All women of reproductive age in Uganda were considered as a source population whereas, the study population included the selected women of reproductive age in the households located in the primary sampling unit or 696 enumeration areas (EAs).

In the 2016 UDHS, multistage-stratified sampling method was used. First, the primary sampling units (PSUs), 697 (162 urban and 535 rural) EAs were selected with probability proportional to size (PPS) within each stratum. The 2014 Uganda National Population and Housing Census was used as a sampling frame after the urban rural stratification was done. The PSUs are typically census enumeration areas. The PSU forms the survey cluster. Clusters are groupings of selected households (30 per EA or EA segment) that participated in the survey. Since one cluster from Acholi sub-region was eliminated because of land disputes, this study was conducted among 696 EAs.

Second, the secondary sampling unit, 20,880 households (30 per EA or EA segment) was randomly selected from each of the 696 accessible selected EAs. Third, the tertiary sampling unit, 18,506 women were selected randomly from total of 20,880 households. Moreover, individual and community level data consisting of health indicators (fertility, maternal health, and reproductive health), and the data regarding the Global Positioning System (GPS) were used. Then the important datasets were linked with the GPS data with its respective cluster for spatial analysis [17].

### Variables

The outcome interest of this study was women with an unmet need for family planning, whereas, the explanatory (determinant) variables were included after reviewing of literatures [46, 47]. Accordingly, individual level factors like; age, religion, marital status, number of children, age at first marriage, educational status, knowledge of any methods, use of contraceptives, wealth status, living child with the current pregnancy, and exposure to media are among others, and the community level factors such as; residence (urban and rural), region and others were included.

### Operational definitions

#### Unmet need for family planning

It is the percentage of women who are fecund and sexually active but are not using any method of contraception, and do not want any more children (unmet need for limiting) or want to delay the next child (unmet need for spacing), or women whose pregnancies were unwanted or mistimed, or all postpartum amenorrheic women whose last birth was unwanted, or mistimed or all fecund women who are neither pregnant nor postpartum amenorrheic, and who either do not want any more children (limit), or who wish to postpone the birth of a child (spacing) for at least two years, but are not using any contraceptive method [48, 49].

The current measure of unmet need is calculated as follows:

$$\mathrm{Unme}\;\mathrm{tNeed}\;\mathrm{for}\;\mathrm{FP}=\frac{\mathrm{WRA}\;\mathrm{who}\;\mathrm{want}\;\mathrm{to}\;\mathrm{limit}\;\mathrm{or}\;\mathrm{space}\;\mathrm{birth}\;\mathrm{for}\;2+\mathrm{years}\;\mathrm{AND}\;\mathrm{are}\;\mathrm{not}\;\mathrm{using}\;\mathrm{contraception}}{\mathrm{Fecund}\;\mathrm{and}\;\mathrm{sexually}\;\mathrm{active}\;\mathrm{WRA}\;(\mathrm{age}15-49)}$$

For this indicator, the denominator is women who would be at risk of pregnancy and includes women who: (1) are either married or are in a sexual union; (2) report being sexually active; and (3) are fecund, and are therefore at risk of becoming pregnant [50].

#### Met need for contraception

Percentage of women attain their desired number of children and the spacing and timing of their birth using contraception, and who are sterilized, or say they cannot get pregnant when asked about the desire for future children.

#### Total demand for contraception

It is the sum of unmet need for family planning and current contraceptive use (any method).

### Data processing and management

The data processing was done after the permission granted and data accessed from the demography heath survey (DHS) program official database (www.measuredhs.com). Then, we used the Individual Record (IR file) data set and extracted the outcome interest (Unmet need for family planning) and predictor variables. The SPSS Version 26 (www.spss.com), STATA 14.2, ArcGIS 10.8 [51], and SaTScan 10.1.2 [52] software were used to analyze the weighted frequency, multivariable multi-level logistic regression model, spatial analysis and Bernoulli model, respectively. In order to correct for the disproportionality of the sample with respect to the target population of interest, the data were weighted using sampling weight, primary sampling unit and strata for complex sample analysis.

### Statistical analysis

#### Model building for multi-level analysis

The characteristics of study subjects were described using weighted frequencies and percentage. Due to the hierarchical nature of the DHS data, the assumptions of the conventional logistic regression model like independence of observations and equal variance assumption were violated. Because study subjects were nested within households, and households were nested within clusters. Within each cluster, women may have similar characteristics. Hence, the multivariable multi-level binary logistic regression model was used to determine the effect of different predictors with the unmet need for family planning among women of reproductive age. In this study, since the Intraclass Correlation Coefficient (ICC) and/or the random effect of the variance was significant and the ICC greater than zero in the null model indicated us to use multilevel regression model than the standard single-level regression model [53–55]. This led to the fitting of four models where, the first model was constructed without predictor variables to assess the effect of community variation on unmet need for family planning among women of reproductive age. The second model regresses the individual-level factors, while in the third model, community-level factors were included. Finally, in the fourth model both individual-level and community level factors were included in the analysis.

### Parameter estimation method

Both the multivariable multi-level logistic regression model and the generalized linear mixed model (GLMM) are used for analyzing nested data. But since the data nature of the outcome variable was not longitudinal or repeatedly measured, the multivariable multi-level logistic regression model is more appropriate compared to GLMM. Therefore, a multivariable multi-level binary logistic regression model was used to account for the variability between clusters. Therefore, to assess the clustering effect of determinants to the unmet need for family planning, mixed-effect model was fitted with a cluster-level random intercept. It comprises of both fixed and random effect analyses.

### Fixed effect

The relationships and strength between unmet need for family planning and predictors were shown using an adjusted Odds Ratio (AOR) with 95% CIs and a p-value of less than 0.05 in the fixed effects measure of association [56].

$${\text{Log}}\left(\frac{\pi ij}{1-\pi ij}\right)=\beta 0+\beta 1xij+\beta 2xij+\dots uj+eij$$

where, πij: the probability of having unmet need for family planning, 1 − πij: the probability of met need for family planning, and β1xij; are individual and community level variables for the i^th^ individual in group j. The ß’s are fixed coefficients indicating a unit increase in X can cause a ß unit increase in the probability of unmet need for family planning, while the ß0 is the intercept which reflects the effect on unmet need for family planning when the effect of all explanatory variables is absent. The uj shows the random effect (effect of the clusters on the unmet need for family planning) for the j^th^ cluster. Due to the nature of DHS data, the within and between cluster variations were taken into account assuming each cluster has a fixed coefficient (β) and a different intercept (β0) [56, 57].

### Random effect

The measure of variation between clusters in the multi-level models was estimated by the Intra Class Correlation Coefficient (ICC) or (variance partition coefficient), Median Odds Ratio (MOR), and Proportional Change in Variance (PCV). The ICC is calculated as; $ICC=\frac{{\text{VA}}}{{\text{VA}}+3.29}*100\%$ or $ICC=\frac{{\text{VA}}}{{\text{VA}}+\frac{{\pi }^{2}}{3}}*100\%$, where VA is area level variance of the model. The Median Odds Ratio is defined as the median value of the odds ratio of unmet need for family planning between the area at the highest risk and the area at the lowest risk among clusters or it quantify unexplained cluster variability (heterogeneity). The MOR is calculated as $MOR={\text{exp}}.[\sqrt{\left(2*VA\right)}*0.6745]$ or ${MOR=e}^{{0.95}^{\sqrt{VA}}}$. The Proportional Change in Variance measures the total variation of unmet need for family planning among women of reproductive age explained by individual and community level factors. The PCV is calculated as; $PCV=\frac{Vnull-VA}{Vnull}*100\%$, where; Vnull = variance of the null model, and VA = area level variance of the model [56, 57].

Mixed effect model with the highest likelihood and the lowest deviance, and the lowest Information Criteria (AIC and BIC) (model 4) was considered the best fit model. There was no multicollinearity between predictors of unmet need for family planning in all models based on the Variance Inflation Factors (VIF) results, since the VIF value of all variables is lower than 10 and tolerance greater than 0.1.

### Spatial analysis

#### Spatial autocorrelation analysis

First, the subnational boundaries of administrative regions of Uganda were obtained from the DHS program official website (https://spatialdata.dhsprogram.com/home/). Then, the spatial analysis was conducted after the occurrence of unmet need for family planning from demographic health survey data was joined and related with each cluster to the corresponding geospatial location using ArcGIS 10.8 software.

The foundation for the spatial autocorrelation was the first Law of Geography, and according to Waldo Tobler, it is “Everything is related to everything else, however nearest things are related than distant things [58].”

Spatial autocorrelation refers to a measure of similarity (correlation) between nearby observations of variables. In this study, Global Moran’s Index (Moran’s I) inferential statistic was used to check for spatial autocorrelation. Its’ value ranges from -1 to 1. The statistically significant Moran’s I (*p* < 0.05), positive z-score, and Moran’s I above zero leads to the rejection of the null hypothesis (unmet need for family planning among women is randomly distributed) and the adjacent observations are more spatially clustered with similar data values (disease/event clustered) (high-high or low-low), then it indicates the existence of positive spatial autocorrelation. The statistically significant Moran’s I (*p* < 0.05), negative z-score and Moran’s I below zero leads to the rejection of the null hypothesis, and indicates that the adjacent observations more spatially dispersed (different values of disease/event clustered together)(high-low or low–high), then it indicates existence of negative spatial autocorrelation [59–61].

The global Moran’s I expressed as follows:

$$I=n\frac{\sum_{i=1}^{n}\sum_{j=1}^{n}{w}_{ij}({x}_{i}-\overline{{\text{x}} })({x}_{j}-\overline{{\text{x}} })}{\sum_{i=1}^{n}{w}_{ij}({x}_{i}-\overline{{\text{x}} })}$$

Where *n* is the number of observations in the whole cluster, *x*i and *x*j are the observations at locations of i and j, W_*ij*_ is the spatial weights between location *i* and* j*, and x̄ is the mean of × [62].

### Hot spot analysis

Hotspot analysis is a spatial analysis and mapping technique used to identify the clustering of spatial phenomena. Accordingly, in this study, hotspot analysis was made by computing Gettis-OrdGi* statistics to identify geographic areas with high clusters and low clusters. The output with statistically significant high or positive GI* score indicates clustering of high values or “hotspot” (occurrence of high prevalence rate of unmet need for family planning), whereas statistically significant low or negative GI*score indicates clustering of low values or a “cold spot” (occurrence of low prevalence rate of unmet need for family planning) [63].

### Spatial interpolation

It is the process of using sampled EAs with known values of unmet need for family planning to estimate the unmet need for FP on the un-sampled areas of the country. In this study, one of the most basic of Kriging methods which is Ordinary Kriging spatial interpolation method [64] was used to predict the prevalence of the unmet need for family planning in unobserved areas of the country.

### Spatial scan statistical analysis

The circular spatial scan statistics is a widely used statistical method for the automatic detection of disease /event clusters from the data by using a moving, varying diameter window to evaluate clusters across all the study area. In this study, Kuldorff’s SaTScan v 10.1.2 software was used to detect the presence of statistically significant spatial clusters of unmet need for FP. Then, the software calculates; the number of observed and expected observations and the likelihood function for each window location and size [65, 66]. Bernoulli model was applied by using geographic coordinates and fitted by considering women with unmet need as cases and women with met need as controls. The default maximum spatial cluster size of 50% of the population at risk (percent cases in area) was used to detect the small and large clusters and clusters with more than this maximum limit was ignored. The primary, secondary, tertiary, and the fourth clusters were identified and ranked based on their likelihood ratio and its significant p-value. Therefore, areas with high Log Likelihood Ratio and significant p-value were considered as high in unmet need for family planning compared to areas outside of the window. The likelihood function for the Bernoulli model is:

$$\text{LLR}=\left(\frac cn\right)^c\left(\frac{n-c}n\right)^{n-c}\left(\frac{C-c}{N-n}\right)^{C-c}\left(\frac{(N-n)-(C-c)}{N-n}\right)^{(N-n)-(C-c)},$$

Where C is the total number of cases, c is the observed number of cases within the window, n is the total number of cases and controls within the window, N is the combined total of cases and controls within the data set [67].

### Patient and public involvement

This study used a publicly available data set (UDHS 2016); therefore, no patients and/or the public involvement in the design, or conduct, or reporting or dissemination plans of this research.

## Results

### Socio‑demographic characteristics of women

In this study, a total of 18,506 women from 696 clusters nested in 15 regions were included. Among these respondent, 23.0% and 20.7% of women were in the age range of 15–19 years and 20–24 years, respectively. The majority of participants lived in a rural area (73.3%). Of the total study participants, more than half, 57.4% of them were attended primary school, more than one-third, 39.6% of them were followers of Catholic religion, 13.5% of them were from South Buganda region, and about one-third, 35.9% of the women were from low-income households in terms of household wealth status. More than half, 60.6% of the respondents were married or living with a partner (Table 1).

**Table 1:** Socio-demographic characteristics of reproductive age women in Uganda, UDHS 2016 (*n* = 18,506)

Variables	Weighted frequency	Weighted percent
Age		
15–19	4,264	23.0
20–24	3,822	20.7
25–29	3,051	16.5
30–34	2,543	13.7
35–39	2,011	10.9
40–44	1,608	8.7
45–49	1,207	6.5
Place of residence		
Urban	4,943	26.7
Rural	13,563	73.3
Region		
Kampala	1,025	5.5
South Buganda	2,494	13.5
North Buganda	1,963	10.6
Busoga	1,690	9.1
Bukedi	1,169	6.3
Bugisu	921	5.0
Teso	1,099	5.9
Karamoja	365	2.0
Lango	1,010	5.5
Acholi	924	5.0
West Nile	1,247	6.7
Bunyoro	1,014	5.5
Tooro	1,357	7.3
Ankole	1,498	8.1
Kigezi	732	4.0
Educational status of women		
No education	1,781	9.6
Primary	10,630	57.4
Secondary	4,639	25.1
Higher	1,456	7.9
Religion		
Anglican	5,774	31.2
Catholic	7,335	39.6
Muslim	2,388	12.9
Seventh Day Adventist	305	1.6
Pentecostal/Born Again/Evangelical	2,468	13.3
Others^a^	236	1.3
Marital status		
Never In Union	4,783	25.8
Married	5,614	30.3
Living with partner	5,609	30.3
Widowed, Divorced, Separated	2,500	13.5
Wealth status		
Poor	6,643	35.9
Middle	3,460	18.7
High	8,403	45.4

^a^Baha'i, Baptist, Presbyterian, Mammon, Jehovah's Witness, Salvation Army, Traditional, Orthodox, and No religion

### Family planning related characteristics of women

Of the total study participants, almost all, 99.0% of them knew about any family planning method, and one fourth, 25.6% of them were visited by field workers within 12 months. Of the total respondents who were visited by field workers within 12 months, nearly one third, 30.8% of them were told by fieldworker about family planning. Among the total participants, 68.6% of them were visited health facility within 12 months, and 68.7% of them had exposure to media (Radio, TV, magazine, news, text) (Table 2).

**Table 2:** Contraceptive related characteristics of women of reproductive age in Uganda, UDHS 2016

Variables	Weighted frequency	Weighted percent
Knowledge of any methods		
No	187	1.0
Yes	18,319	99.0
Visited by field workers within 12 months		
No	13,762	74.4
Yes	4,744	25.6
Told by fieldworker about family planning (*n* = 4744)		
No	3,281	69.2
Yes	1,463	30.8
Visited health facility within 12 months		
No	5,814	31.4
Yes	12,692	68.6
Told of family planning method at health facility (*n* = 12,692)		
No	7,762	61.2
Yes	4,930	38.8
Exposure to media (Radio, TV, magazine, news, text)		
No	5,795	31.3
Yes	12,711	68.7

### Prevalence and distribution of unmet need for family planning across different socio-demographic

In this study, the magnitude of unmet need for family planning among fecund and sexually active reproductive-age women was 27.7% (95% CI 27, 29). Of the total unmet need for family planning, majority, 80.5% of unmet need were seen among rural residents, 12.0% and 10.0% of unmet need were seen among participants from Busoga and West Nile, and 64.2% of unmet need seen among participants attended primary education (Table 3).

**Table 3:** The variation of unmet need for family planning across different socio-demographic related characteristics of women of reproductive age in Uganda, UDHS 2016

Variable		Family planning need	
		**Unmet**	**Met**
		**Weighted Frequency (%)**	**Weighted Frequency (%)**
Place of residence	Urban	650(19.5)	2,451(28.1)
	Rural	2,689(80.5)	6,261(71.9)
Region	Kampala	135(4.0)	467(5.4)
	South Buganda	303(9.1)	1,305(15.0)
	North Buganda	302(9.0)	1,010(11.6)
	Busoga	401(12.0)	726(8.3)
	Bukedi	238(7.1)	587(6.7)
	Bugisu	167(5.0)	459(5.3)
	Teso	250(7.5)	456(5.2)
	Karamoja	49(1.5)	204(2.3)
	Lango	200(6.0)	471(5.4)
	Acholi	225(6.7)	340(3.9)
	West Nile	335(10.0)	405(4.6)
	Bunyoro	187(5.6)	470(5.4)
	Tooro	237(7.1)	685(7.9)
	Ankole	221(6.6)	761(8.7)
	Kigezi	89(2.7)	366(4.2)
Highest educational status	No education	436(13.1)	774(8.9)
	Primary	2,144(64.2)	4,873(55.9)
	Secondary	577(17.3)	2,242(25.7)
	Higher	181(5.4)	823(9.4)
Wealth Index	Poor	1,549(46.4)	2,936(33.7)
	Middle	668(20.0)	1,623(18.6)
	High	1,121(33.6)	4,154(47.7)

### Factors associated with unmet need family planning

#### Multi‑level logistic regression analysis (Random effect analysis)

The ICC result based on estimated intercept component variance indicated that about 6.34% of the variance in the odds of unmet need for family planning among WRA could be explained by between-cluster differences. After adjusting for individual-level and community-level factors, the variation in unmet need for family planning across different clusters remained statistically significant. In the final model (Model IV), as indicated by the PCV, the effect of clustering is still statistically significant, 84% of the variation in unmet need for FP across communities was explained by both individual and community level factors.

Moreover, the median odds ratio between the higher and lower-risk areas of unmet need for family planning among clusters was 1.56 in the null model, showed that there was variation between communities (clustering) (56% times higher than the reference (MOR = 1)). It also reflects that during random selection of two clusters, women from clusters of high risk of unmet need for FP had 1.56 times more likely to experience an unmet need for family planning than women at the cluster with low risks of unmet need.

Moreover, model fitness was also checked using deviance and log likelihood test. Accordingly, model IV with the lowest deviance (13,109.52) and highest log likelihood (-6554.76) was used to identify significantly associated factors with unmet need for FP. Therefore, all interpretations and conclusions were made based on the fitted model, model IV **(**Table 4**)**.

**Table 4:** Multivariable multilevel logistic regression analysis result of both individual and community-level factors associated with unmet need for family planning in Uganda, UDHS 2016

Individual and community level characteristics	Null-Model (Model I)	Model II (Individual-level factors)	Model III (Community-level factors)	Model IV (Both Individual and community-level Factors)
		**AOR (95% CI)**	**AOR (95% CI)**	**AOR (95% CI)**
Place of residence				
Urban			1	1
Rural			1.38(1.21, 1.58)	1.05(0.92, 1.21)
Region				
Kampala			1	1
South Buganda			0.745(0.57, 0.98)	0.72(0.55, 0.95)*
North Buganda			0.95(0.73, 1.25)	0.83(0.63, 1.09)
Busoga			1.56(1.19, 2.04)	1.28(0.98, 1.69)
Bukedi			1.15(0.86, 1.52)	0.92(0.69, 1.23)
Bugisu			1.02(0.76, 1.37)	0.86(0.64, 1.17)
Teso			1.59(1.20, 2.10)	1.27(0.95, 1.70)
Karamoja			0.71(0.51, 0.98)	0.46(0.33, 0.65)***
Lango			1.15(0.86, 1.53)	0.86(0.64, 1.16)
Acholi			2.04(1.54, 2.70)	1.49(1.11, 1.99)**
West Nile			2.43(1.84, 3.20)	1.86(1.39, 2.47)***
Bunyoro			1.13(0.85, 1.49)	0.88(0.66, 1.18)
Tooro			1.01(0.76, 1.34)	0.85(0.64, 1.14)
Ankole			0.85(0.64, 1.13)	0.71(0.53, 0.95)*
Kigezi			0.71(0.52 0.96)	0.64(0.46, 0.88)**
Age of the respondents				
15–19 years		1		1
20–24 years		0.93(0.79, 1.11)		0.94(0.79, 1.11)
25–49 years		0.84(0.71, 0.99)		0.84(0.72, 0.99)*
Educational status of women				
No education		1		1
Primary		0.92(0.81, 1.05)		0.81(0.71, 0.93)**
Secondary		0.81(0.68, 0.96)		0.71(0.59, 0.84)***
Higher		0.80(0.63, 1.02)		0.69(0.54, 0.88)**
Religion				
Anglican		1		1
Catholic		1.13(1.01, 1.23)		1.10(0.99, 1.23)
Muslim		1.34(1.16, 1.56)		1.20(1.03, 1.39)*
Seventh Day Adventist		1.01(0.71, 1.44)		1.06(0.75, 1.51)
Pentecostal/Born Again/Evangelical		1.19(1.04, 1.38)		1.21(1.05, 1.39)**
Other^+^		1.24(0.87, 1.78)		1.27(0.89, 1.80)
Wealth status				
Poor		1		1
Middle		0.85(0.76, 0.96)		0.93(0.83, 1.05)
High		0.64(0.57, 0.72)		0.73(0.64, 0.82)***
Exposure to media (Radio, TV, magazine, news, text)				
No		1		1
Yes		0.91(0.82, 0.99)		0.92(0.84, 1.01)
Living child with the current pregnancy				
< 5		1		1
> = 5		1.75(1.57, 1.95)		1.69(1.51, 1.88)***
Age at first marriage				
< 15 years		1		1
15–24 years		0.99(0.89, 1.10)		1.01(0.91, 1.13)
> 24 years		0.99(0.82, 1.20)		1.03(0.85, 1.25)
Random effects				
Community level variance with (SE)	0.222(0.028)	0.126(0.058)	0.066(0.019)	0.035(0.018)
*p*-value	< 0.0001	< 0.0001	< 0.0001	< 0.0001
ICC (%)	6.34	3.69	1.98	1.05
PCV-Explained variation	Reference	0.43	0.70	0.84
MOR	1.56	1.40	1.28	1.19
Model Fitness				
Log-likelihood (LL)	-7179.43	-6641.73	-7055.98	-6554.76
DIC (-2LL)	14,358.86	13,283.46	14,111.96	13,109.52
AIC	14,362.86	13,319.46	14,145.96	13,175.52
BIC	14,377.67	13,451.51	14,271.81	13,417.61
Multicollinearity				
VIF		1.12	1.00	1.16

Key: *AOR* Adjusted Odds Ratio, *CI* Confidence interval, *1* reference group, *ICC* Intracluster Correlation Coefficient, *MOR* Median Odds Ratio, *PCV* Proportional change in variance, *DIC* Deviance information criterion, *AIC* Akaike Information Criteria, *BIC* Bayesian Information Criteria, *VIF* Variance inflation factor, *: *p* < 0.05; **: *p* < 0.01; ***: *p* < 0.001, and ^+^: Baha'i, Baptist, Presbyterian, Mammon, Jehovah's Witness, Salvation Army, Traditional, Orthodox, and No religion.

### Multi‑level logistic regression analysis (Fixed effect analysis)

Using multivariable multi-level logistic regression analysis different individual-level factors like; age of the respondents, educational status of women, religion, wealth status, exposure to media, and living child with the current pregnancy were significantly associated factors with unmet need, whereas community- level factors like; place of residence, and region were also found to be significantly associated predictors with unmet need for FP.

After all individual and community level factors were controlled for potential confounders using multivariable analysis, factors such as; region, age of the respondents, educational status of women, religion, wealth status, and living child with the current pregnancy were the strong predictors for the unmet need for family planning method among WRA.

The odds of having unmet need for FP among WRA in North Buganda, Busoga, Bukedi, Bugisu, Tooro, Bunyoro, and Teso was not significantly different from those having unmet in Kampala. The odds of having unmet need for FP among women in the South Buganda (AOR = 0.72, 95% CI: 0.55, 0.95), Karamoja (AOR = 0.46, 95% CI: 0.33, 0.65), Ankole (AOR = 0.71, 95% CI: 0.53, 0.95), and Kigezi (AOR = 0.64, 95% CI: 0.46, 0.88) were lower by 28%, 54%, 29%, and 36% compared to women in Kampala Region, respectively. In contrast to this, the odds of having unmet need for FP among women in the Acholi (AOR = 1.49, 95% CI: 1.11, 1.99) and West Nile (AOR = 1.86, 95% CI: 1.39, 2.47) was higher by 49%, and 86% compared to women in Kampala Region, respectively.

Women aged 25–49 years old were 16% less likely to have unmet need for FP compared with women in the youngest age group (15–19 years old) (AOR = 0.84; 95% CI 0.72, 0.99). Those women who attended primary (AOR = 0.81; 95% CI 0.71, 0.93), secondary (AOR = 0.71; 95% CI 0.59, 0.84), and higher level education (AOR = 0.69; 95% CI 0.54, 0.88) were 19%, 29%, and 31% less likely to have unmet need for FP compared to women with no education, respectively.

The odds of having unmet need for FP among Muslim (AOR = 1.20, 95% CI: 1.03, 1.39) and Pentecostal/Born Again/Evangelical (AOR = 1.21, 95% CI: 1.05, 1.39) religion followers were higher by 20%, and 21% compared to those women who are Anglican religion followers, respectively.

The odds of having unmet need for FP among women with high wealth status was lower by 27% (AOR = 0.73, 95% CI: 0.64, 0.82) compared to those women with lowest wealth quintiles. Moreover, the odds of having unmet need among women who had >  = 5 living child with the current pregnancy was higher by 69% compared to those women who had less than five children (AOR = 1.69, 95% CI: 1.51, 1.88) (Table 4).

### Spatial data analysis

**Fig. 2:**
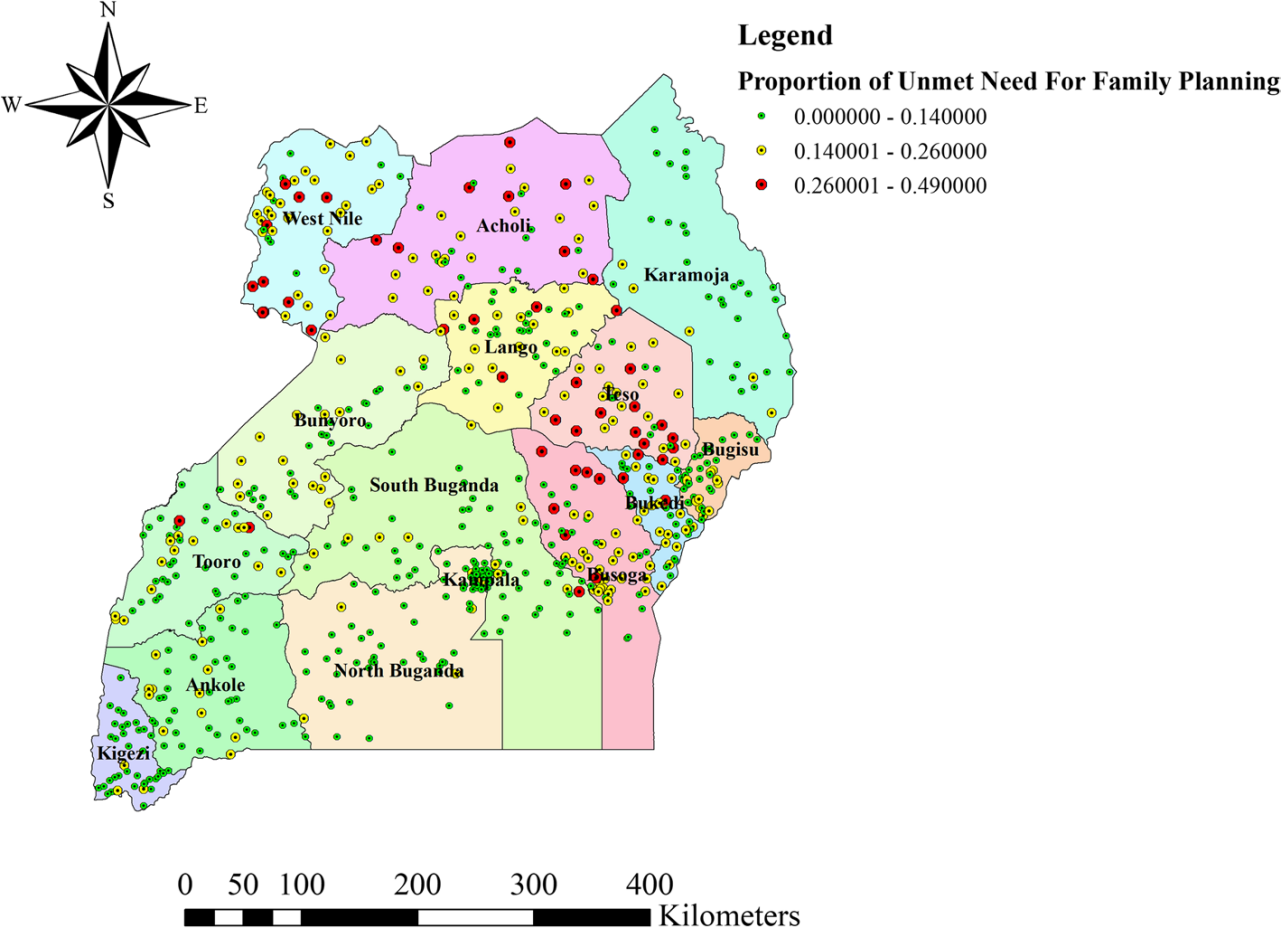
Spatial distribution of unmet need for family planning among women of reproductive age, Uganda, 2016

A total of 685 clusters were considered for spatial analysis of the unmet need for family planning among women of reproductive age, the remaining 11 clusters were excluded due to having 0 coordinates. This is due to clusters with zero coordinates couldn’t even be treated as a systematically displaced coordinates because the difference compared to the true point is greater than 1%. From the total number of eligible women who had unmet need for family planning, there were only about 44 (1.26%) of households (HHs) with eligible women who had unmet need for family planning had 0 coordinates. Therefore, analyzing of the data with clusters of zero coordinate will give us a wrong spatial interpolation and geographic location for hotspot analysis because Uganda is located in southeast Africa between 1º S and 4º N latitude, and between 30º E and 35º E longitude. Accordingly, a higher proportion of unmet need for family planning occurred in Northern Busoga, Central and Southern Teso, Northern and Eastern parts of Acholi, and West and Southwest parts of West Nile **(**Fig. 2**)**.

### Spatial autocorrelation

**Fig. 3:**
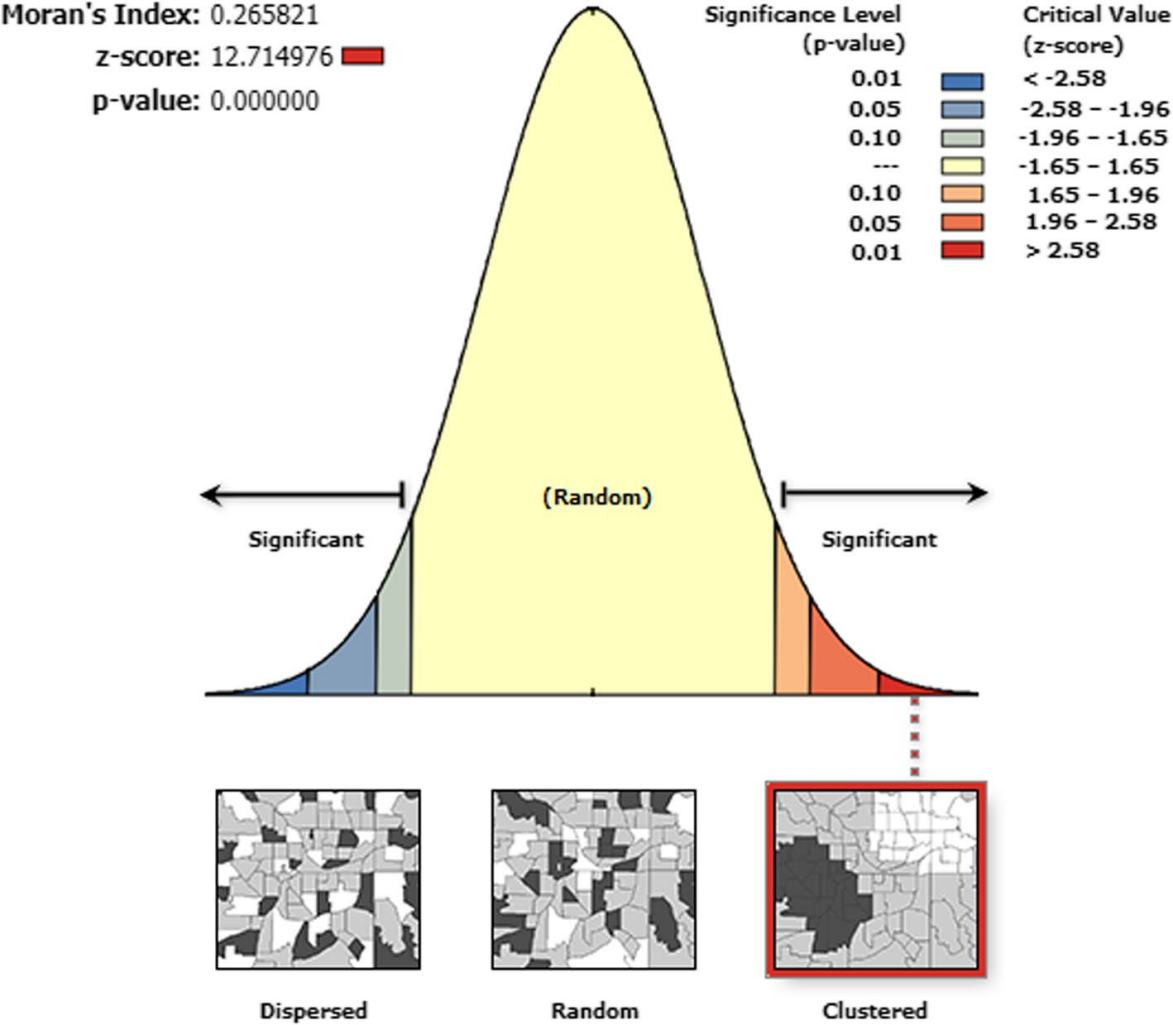
Spatial autocorrelation analysis of unmet need for family planning among women of reproductive age, Uganda, 2016

In this study, the analysis of spatial autocorrelation indicated that the spatial distribution of unmet need for family planning was nonrandom (alternative hypotheses is accepted) in Uganda. The Global Moran’s I value of 0.27 (*p*-value < 0.0001) and the Z-score of 12.71 indicated that there was significant clustering of unmet need for family planning in the study area **(**Fig. 3**)**.

### Hot spot analysis of unmet need for family planning

**Fig. 4:**
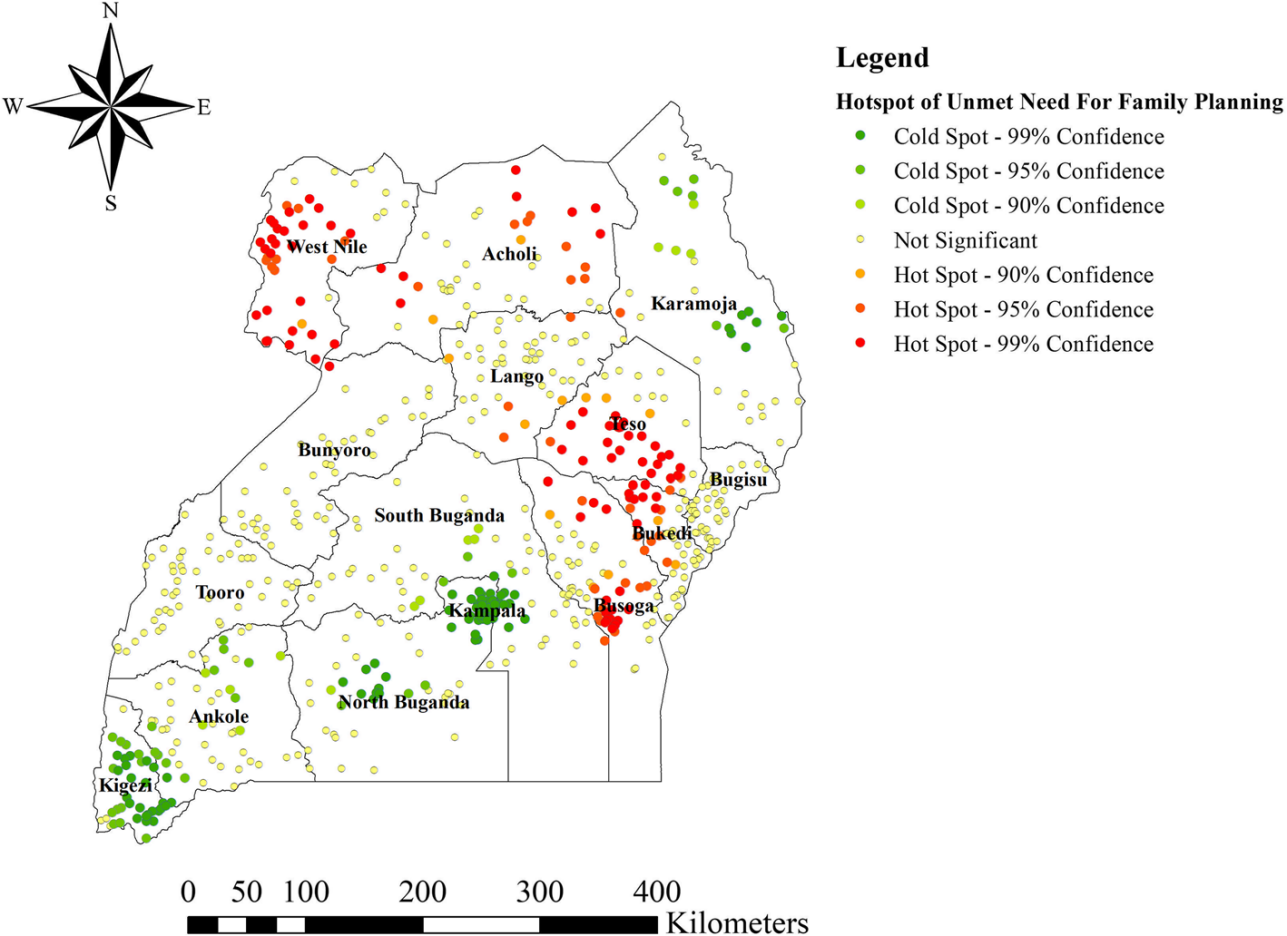
Hot spot analysis of unmet need for family planning among women of reproductive age, Uganda, 2016

Based on Getis OrdGi statistical analysis method, this study identified significant hotspot areas (high rate of unmet need for FP) in West and South Western part of Teso, Northern and Northwest part of Bukedi, Central part of Busoga, Western and South Western part of West Nile, and Eastern part of Acholi, whereas the cold spot areas (low-risk unmet need for FP) were also seen in Eastern and Southeastern parts of Kigezi, Central part of North Buganda, Kampala, and northern and eastern parts of Karamoja (Fig. 4).

### Spatial interpolation of unmet need for family planning in Uganda

**Fig. 5:**
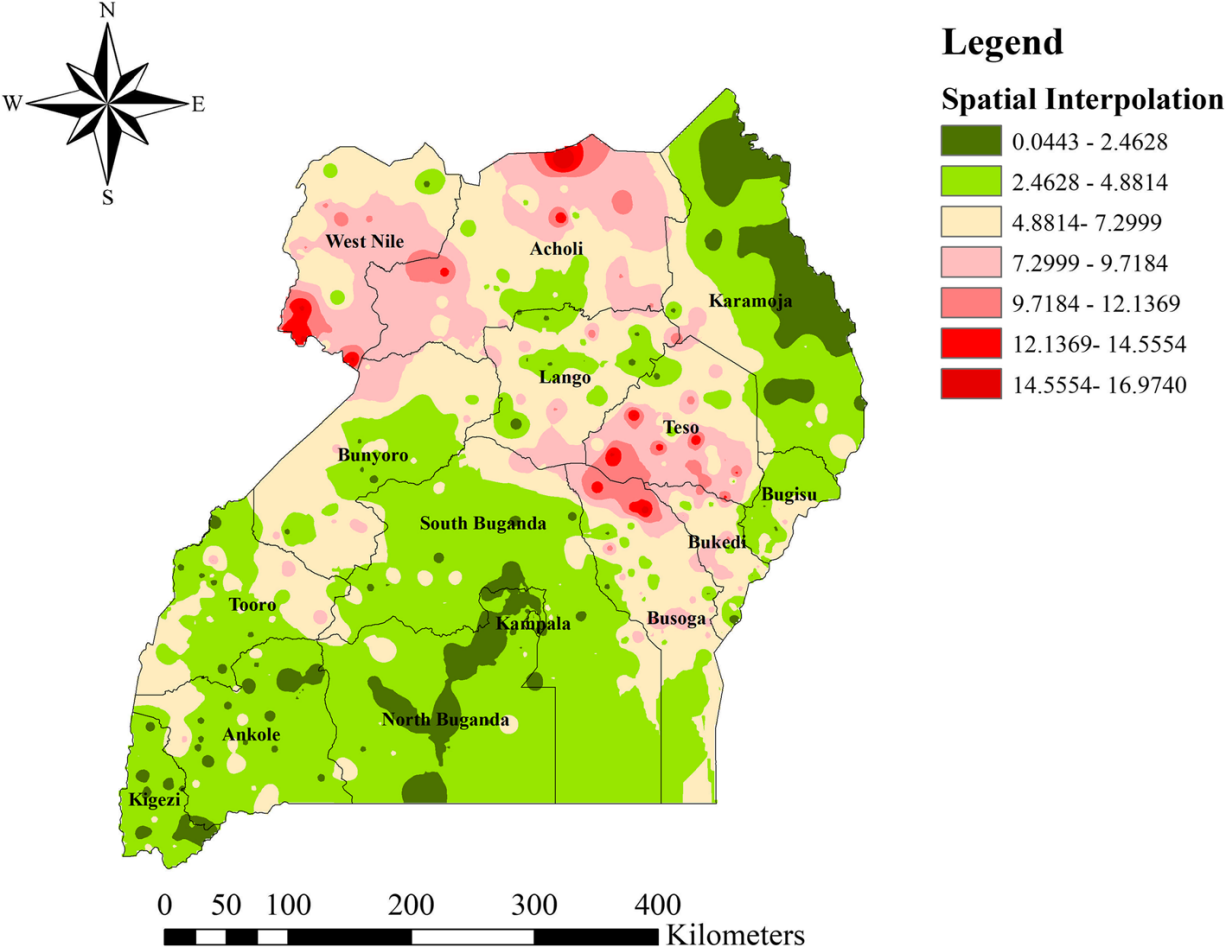
Spatial Interpolation of unmet need for family planning among reproductive age group women, Uganda, 2016

The raster surface of Uganda as a result of spatial Interpolation analysis, the reproductive age women in Northern and eastern part of Acholi, South and South Western part of West Nile, Southwestern part of Teso, and Northern part of Busoga regions are predicted to have a more unmet need for family planning than women residing in other areas. In contrast to this, women in Northern, North Eastern and Eastern part of Karamoja, Southern part of Kampala, Northern, Central and Southern part of North Buganda, and South Eastern part of Kigezi regions are predicted to have a less unmet need for family planning than women residing in other areas (Fig. 5).

### Spatial SaTScan analysis of the unmet need for family planning

Purely Spatial SaTScan analysis was employed for scanning of clusters with high rates using the Bernoulli model and recognized a total of 178 significant clusters. Of which, 80 primary clusters (most likely), 44 secondary clusters, 38 tertiary clusters, and 16 quaternary cluster of unmet need for FP were identified.

**Fig. 6:**
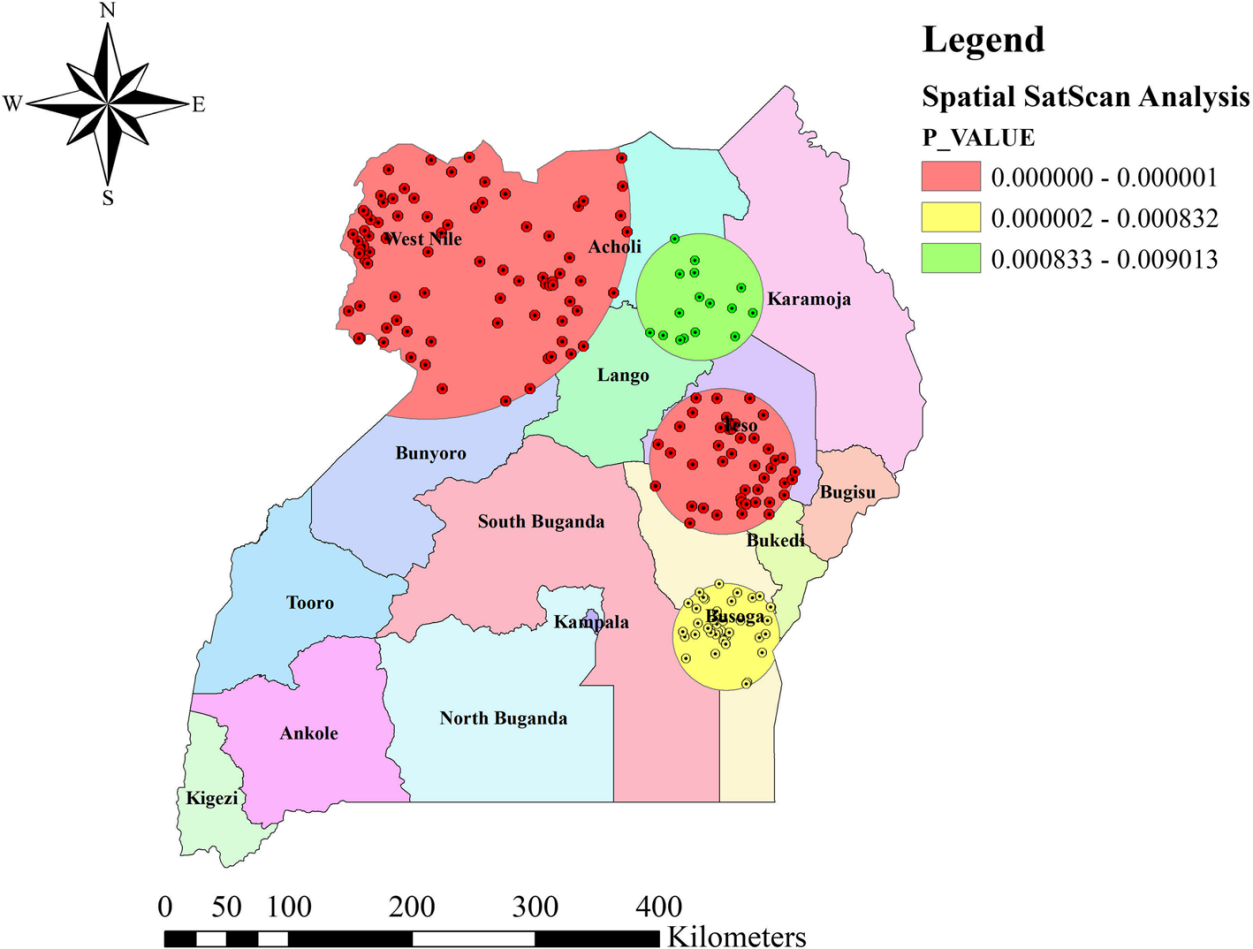
Spatial SaTScan analysis of unmet need for family planning among reproductive age group women, Uganda, 2016

The primary clusters were located in the West Nile, Western part of Acholi, and Northern part of Bunyoro regions at 3.268179 N and 31.393413 E coordinate, and 164.18 km radius. This cluster has a Relative Risk (RR) of 1.62, and Log-Likelihood Ratio (LLR) of 78.11, at *p*-value of < 0.0001. This indicated that women of reproductive age within the spatial window had 1.62 times more likely to experience the unmet need for family planning as compared to women outside the spatial window. The secondary clusters were found in Tuso, Northern part of Bukedi and Northern part of Busoga regions at coordinate of 1.484156 N and 33.543849 E, with 59.11 km radius. The risk of experiencing unmet need for FP among WRA in this window is 1.40 times more likely to have unmet needs than women outside this window (RR = 1.40, LLR = 25.6, p-value < 0.0001). The tertiary clusters’ spatial window was typically located in the Central part of the Busoga region at 0.204769 N, and 33.569889 E, with 43.30 km radius (RR = 1.36, LLR = 14.2, *p*-value < 0.001). The quaternary clusters’ spatial window sited in South Eastern part of Acholi, Western part of Karamoja, and North eastern part of Lango regions at 2.681019 N, and 33.376881 E, with 51.38 km radius. Women within the spatial window had 1.49 times more likely to experience unmet need for family planning as compared to women outside the spatial window (RR = 1.49, LLR = 11.44, *p*-value < 0.01) (Table 5, Fig. 6**)**.

**Table 5:** SaTScan analysis of the unmet need for family planning among reproductive age group women in Uganda, 2016

Clusters Detected	Coordinate/ radius	No_ of Clusters	Population	Number cases	Expected cases	RR	Percent cases in areas	LLR	*p*-value
1st	(3.268179 N, 31.393413 E) / 164.18 km	80	1409	609	403	1.62	43.2	78.11	< 0.0001
2nd	(1.484156 N, 33.543849 E) / 59.11 km	44	965	375	276	1.40	38.9	25.60	< 0.0001
3rd	(0.204769 N, 33.569889 E) / 43.30 km	38	631	241	180	1.36	38.2	14.2	< 0.001
4th	(2.681019 N, 33.376881 E) / 51.38 km	16	271	114	77	1.49	42.1	11.44	< 0.01

NB: *LLR* Log-Likelihood Ratio, *RR* Relative Risk.

## Discussion

One of the 2030 sustainable development goals is to achieve the universal access to reproductive health services [68]. Therefore, to achieve the desired goal by the end of 2030, problems related to the burden of unmet need for family planning and its spatial distribution should be identified and intervened at each countries of the world as per the result. Thus, this study is aimed to assess the spatial distribution and determinants of unmet need for family planning among WRA in Uganda.

### General findings

In this study, the magnitude of unmet need for family planning among reproductive-age women was 27.7% (95% CI 27, 29). In multivariable mixed-effect logistic regression analysis (Model IV); factors such as; age of the respondents, educational status of women, religion, wealth status, living child with the current pregnancy, and regional variation were the strong predictors for the unmet need for family planning method among WRA. This study also identified different significant hotspot areas (high rate of unmet need for FP) in West and South Western part of Teso, Northern and Northwest part of Bukedi, Central part of Busoga, Western and South Western part of West Nile, and Eastern part of Acholi regions.

### Comparisons with studies

The present study revealed that the spatial distribution of unmet need for family planning was significantly varied across different regions of the country; where these significant hotspot areas of unmet need for family planning were West and South Western part of Teso, Northern and Northwest part of Bukedi, Central part of Busoga, Western and South Western part of West Nile, and Eastern part of Acholi regions. Similarly different studies related to the spatial distribution of unmet need for family planning in Nigera [69] and Ethiopia [47] also revealed that there are different significant geographical variations among regions. Therefore, the spatial variation with in the regions of Uganda might be due to the difference in socioeconomic status [70], the availability of infrastructures [71], and health care services [72]. For instance, the total fertility rate (TFR) of woman was 6 in Teso and West Nile, and 6.1 in Bukedi and Busoga, which was higher than the national estimate of 5.4, and this might be an indication for high unmet need for FP compared to the other region of the country like Kampala with TFR of 3.5 [17]. Particularly, women from the Central part of Busoga like Jinja and Mayuge districts, the high unmet needs for FP were attributed due to fear of side effects and contraceptive cost, commodity stock outs, negative health care provider attitudes, and poor youth friendly service utilization [73].

The final model of this study also revealed that the regions like West Nile and Acholi has 86% and 49% high unmet need compared to Kampala, respectively. This might be due to various reasons such as; socio-cultural, religious and economic factors, often hinder most women from making informed decisions [74]. Moreover, this could be attributed to the reason that the poverty rates of some regions of the country, such as; 78% in Bukedi, 72% in Busoga, 50% in Teso, 69% in Acholi, and 76% in West Nile regions were higher than the national average, 47% [75], and this could contribute to the poor utilization of the health service (family planning) by the women. The high unmet need for family planning in West Nile and Acholi could also contributed for the high rates of teenage pregnancy which was 22.4% in West Nile and 23.8% in Acholi compared to 16.8% in Kampala [17]. This might be due to the fact that women from rural districts and regions located far from the capital Kampala had poor health care seeking behavior [76].

The high in unmet need for FP in West Nile and Acholi compared to Kampala is also supported by the 2016 UDHS finding that 39.4% of women used modern family planning methods in Kampala whereas only 19.0% of women in West Nile and 30.2% of women in Acholi were used modern family planning methods [17].

In the final model of this study, both individual-level and community-level factors were responsible for about 84% of the disparity of rates of unmet need for family planning among women of reproductive age in Uganda. After adjusting for both individual-level and community-level factors, the magnitude of unmet need for family planning among women was 27.7%. This finding is higher compared to the studies conducted in Ethiopia 15% [47], in Gambia 17.68% [77], in Malawi 21% [78], and in East Africa 20.68% [57]. On the contrary, the magnitude of the current study is lower compared with the previous studies reported that the magnitude of unmet need for FP was 39% in India [79], 32.6% in Saudi Arabia [26], and 51.7% in Angola [80]. This discrepancy might be due to the difference in the study area, health services coverage, sample size, study subject, study period, and the difference in socio-demographic and cultural factors. For instance, the study conducted in Angola only focused on married women, and therefore, married women are more prone to be sexually active and also this in turn will contribute for the unmet need for FP.

Age is an important predictor for unmet need for FP among women. The odds of experiencing unmet need for FP among women aged 25–49 years old were 16% less likely compared with women in the youngest age group 15–19 years. This study is consistent with the studies conducted in different countries of the world [81], in sub Saharan Africa [82], in Bangladish [83], and in Ethiopia [84]. This might be due to the fact that older women are more experienced, educated, matured and can able to decide on different health services including the use of family planning services compared to younger women. Moreover, older woman might have the desired numbers of children compared to younger women, which enforce the woman to use family planning methods. Furthermore, adolescents also experience stigma from providers, have less access to money, and may have less access to information on FP [85].

In this study, those women who attended higher level education were 31% less likely to have unmet need for FP compared to the women with no formal education. This study was comparable with the studies conducted in sub Saharan Africa [86], and in Pakistan [25]. This could be explained by the reason that women with no formal education might not have a chance to decide regarding FP services and are more economically dependent and therefore, these reasons might hinder the use of family planning. Besides, educated women might have good job opportunity, good monthly income, good understandability of importance of health services, and capability of decision about the use of FP. So that the risk of unmet need among educated women become less likely [87].

The higher odds of unmet need for family planning was observed among women who are Muslim and Pentecostal religion followers compared to women who are Anglican religion follower. This study is supported by the studies conducted in different countries like in Nepal [88], and in Uganda [89], where Muslim and Pentecostal belief was positively associated with an increase in unmet need. In contrast to this, study conducted in Ethiopia [90] revealed that Muslim women had lower odds of unmet need. Other study conducted in Ghana [91] revealed that Pentecostal Protestants women had higher odds of unmet need. This discrepancies and similarities of the odds of unmet need for family planning among women with different religion might be explained by the reason that the influence of religious beliefs on the use of family planning method appeared to be complex, especially in low- and middle-income countries (LMICs) [92]. Different cultural [93], personal, community, religious related factors, and existing policies and regulations could also contribute for the poor sexual and reproductive health (SRH) knowledge and practices [94]. However, some Muslim women accept the use of contraception, some women might believe that it is against their religion and the will of GOD/Allah to decide on the number of children [95].

Wealth index was a determinant factor of unmet need for FP in Uganda. The odds of unmet need for family planning among women with highest wealth quantile were lower compared to women with low wealth quantile. This finding is corroborated by the studies conducted in Burundi [20], Ethiopia [96], Nigeria [69], and Pakistan [25]. This could be explained by the fact that women with good economic status have an opportunity to have access to education, exposure to mass media, and accesses to different health related services like family planning services. Besides, women will adhere to different health services due to recognition of importance of health services [97].

Women who had five or more alive children had high odds of unmet need for family planning. This finding was consistent with the studies conducted in Gambia [19] and Burkina Faso [32]. This might be due to the reason that women with too many children might think that they might not be fertile any more associated with their age and this thought may increase their unmet need for FP. However women who had more children were expected to use family planning, the high odds of unmet need for FP among women with more children might be due to socio-cultural influence [98, 99], poor decision towards FP use, and poor discussion with their partner [100, 101].

### Strengths and limitations

This study has its own strengths. Some of the strengths are; first, this study used the national survey data with a large sample size, which increases its generalizability across all regions of Uganda. Second, different analysis methods such as; multilevel logistics regression analysis and spatial analysis (Hotspot and SatScan analysis) were used to identify factors which contribute for the increment of rate of unmet need and statistically significant areas with a high cluster of unmet needs for FP among WRA, respectively. However, this study has also different limitations that need to be kept in mind while its interpretation. Since UDHS used cross-sectional study design, the cause/effect relationship could not be established. Moreover, since the national demographic health survey was conducted using a questionnaire-based survey, the reliability of the data was relied on the recall ability of the respondents, and therefore, there might be a recall bias. Furthermore, the location of data values was displaced to a random direction and a random distance up to 2 km for urban and up to 5 km for rural areas to ensure for the reduction of disclosure of the respondents; thus, this was the challenge to know the exact location of unmet need for family planning. Finally, due to the presence of clusters with zero coordinates, the reader should cautiously understand the results of spatial analysis that the result is only represents for those clusters with non-zero coordinates.

## Conclusion

This study documented that considerable geographic disparities the rate of unmet need for family planning seen at different regions of the country, Uganda. The significant hotspot areas in unmet need for family planning were observed in the West and South Western part of Teso, Northern and Northwest part of Bukedi, Central part of Busoga, Western and South Western part of West Nile, and Eastern part of Acholi regions of the country, while cold spot areas of unmet need for FP were observed in Kampala, central part of North Bugana, and Kigezi. About 84% of the disparity in unmet need for FP occurrence across different regions (EAs) was regarded as being caused by both individual-level and community-level factors. The increased occurrence (odds) of unmet need for FP among women was attributed to different predictors like; age of the respondents (15–19 years), women with no formal education, religion (Muslim and Pentecostal religion), poor wealth status, women with more than five or more alive children, and regional variation.

Hence, the burden of unmet need for FP should be addressed through implementing different intervention approaches such as; giving more emphasis to improve the socio-economic (the educational and economic) status of women, and sexual and reproductive health service should be addressed for young aged women and in the regions where the cultural and religious belief were deep rooted. The government of Uganda should prioritize the provision of contraceptives for the unmet need hotspot areas of the country (West Nile, Acholi, Bukedi, Busoga, and Teso). In addition to this, further qualitative studies shall be conducted to identify the tangible evidence and properly address the cause behind for the high odds of unmet need, especially among women with more than five or more alive children and Muslim and Pentecostal religion followers. In a country where religious belief dominantly accepted, followers require guidance from religious leaders on many aspects of their lives. Therefore, fostering different programs to participate the religious leaders towards the promotion of SRH education will be very crucial for the implementation of SRH services and involvement of wider community.

## Data Availability

The datasets analyzed during the current study are available in the public domain at (URL: https://dhsprogram.com/data/dataset_admin/index.cfm) upon registration at the DHS program website.

## Abbreviations

AIC: Akakies information criteria
AOR: Adjusted odds ratio
BIC: Bayesian information criteria
CI: Confidence Interval
DHS: Demographic Health Survey
DIC: Deviance Information Criterion
EAs: Enumeration Areas
FP: Family Planning
GLMM: Generalized linear mixed models
ICC: Intra-cluster correlation coefficient
ICPD: International Conference on Population and Development
LBW: Low Birth Weight
LLR: Log-Likelihood Ratio
LMICs: Low- and Middle-Income Countries
LICs: Low-Income Countries
MOR: Median odds ratio
PCV: Proportional Change in Variance
PHC: Population and Health Survey
RR: Relative Risk
SDGs: Sustainable Developmental Goals
SRH: Sexual and Reproductive Health
UDHS: Ugandan Demographic and Health Survey
UNFPA: United Nations Fund for Population Activities
VIF: Variance Inflation Factor
WRA: Women of Reproductive Age
